# Promoting physical activity in children through family-based intervention: protocol of the “Active 1 + FUN” randomized controlled trial

**DOI:** 10.1186/s12889-019-6537-3

**Published:** 2019-02-20

**Authors:** Amy S. Ha, Johan Y. Y. Ng, Chris Lonsdale, David R. Lubans, Florrie F. Ng

**Affiliations:** 1 Department of Sports Science and Physical Education, The Chinese University of Hong Kong, Shatin, Hong Kong; 2 Institute for Positive Psychology and Education, Australian Catholic University, 33 Berry Street, North Sydney, NSW 2060 Australia; 3 Priority Research Centre for Physical Activity and Nutrition, School of Education, University of Newcastle, Callaghan, NSW 2308 Australia; 4 Department of Educational Psychology, The Chinese University of Hong Kong, Shatin, Hong Kong

**Keywords:** Family-based intervention, Family-based physical activity, Fundamental movement skills, Moderate-to-vigorous physical activity, Self-determination theory

## Abstract

**Background:**

Physical activity (PA) is beneficial to people’s physical and psychological health. Physically active children are likely to become active adults; thus, active lifestyles should be promoted from childhood. Parents are crucial for shaping their children’s behaviors, but many lack the knowledge and skills to provide optimal support for PA. The “Active 1 + FUN” intervention was designed to enhance PA of the whole family, and improve parenting methods of both fathers and mothers.

**Methods:**

“Active 1 + FUN” is a family-based intervention designed using the tenets of self-determination theory. The intervention was designed to help parents support their children’s basic psychological needs for competence (by providing informational feedback and optimal challenges), autonomy (by exploring a variety of activities and reducing controlling behaviors), and relatedness (by increasing co-PA between parents and child). The intervention components include interactive workshops, activity sessions, physical activity homework, activity planning consultations, easy sports equipment, and online materials. A randomized controlled trial will be conducted to evaluate the intervention. A target sample of 204 Primary three to five students (8 to 11 years) and their parents will be randomly allocated to the experimental group or a wait-list control group. The experimental group will receive a ten-session intervention which spans approximately six months. The control group will receive intervention one year later. Children’s accelerometer-based leisure-time physical activity (primary outcome) and secondary outcomes will be assessed at baseline, at end of intervention period (6 months after baseline), and at follow-up (12 months after baseline). Qualitative interviews will be conducted to determine effective intervention components from the perspective of children and parents. We hypothesize that the intervention will increase parents’ and children’s activity behaviors and that children will experience higher levels of needs satisfaction with regard to physical activity participation.

**Discussion:**

Physical activity interventions often target children only, but ones that also involve parents may be more beneficial. The “Active 1 + FUN” intervention will be organized and delivered by trained trainers. Consequently, this intervention could be scaled to a larger number of Hong Kong schools in the future and could impact a wider population of schoolchildren.

**Trial registration:**

ANZCTR ACTRN12618001524280. Registered 11 September 2018.

## Background

Physical activity is related to better physical and psychological health outcomes across the lifespan. The benefits of physical activity include low blood lipids, low prevalence of overweight and obesity, high bone mineral density, and few symptoms of depression [1, 2]. Researchers have also found that physical activity behaviors or habits track from childhood to adulthood [3]. Essentially, physically active children and adolescents will likely to be physically active during adulthood. Recent research also suggests that physical activity may improve children’s cognitive functions [4, 5] and academic performance at school [6]. Therefore, it is important to ensure that young people are engaging in sufficient levels of physical activity to support their current and future health status.

The development of competency in a range of fundamental movement skills (FMS) during childhood may help to establish a lifelong commitment to physical activity [7]. FMS include locomotor (e.g., running and jumping), object-control (e.g., throwing and catching) and stability (e.g., balancing and twisting) skills, which are regarded as the “building blocks” for movement and the foundation for participation in sports and physical activities [8, 9]. FMS competency of among schoolchildren is positively associated with various health benefits, namely, greater engagement of physical activity, greater cardiorespiratory endurance, higher self-esteem, and lower risks of overweight and obesity [10, 11]. As such, improving primary schoolchildren’s FMS may increase their engagement in physical activity and enable them to live an active and healthy lifestyle throughout their lifespans.

### Inactivity in children: The Hong Kong context

The World Health Organization [12] has recommended that children engage in at least 60 min of moderate-to-vigorous physical activity (MVPA) daily. Despite the known benefits of physical activity, the majority (85 to 90%) of the children in Hong Kong do not meet these recommended levels of physical activity [13, 14]. In response, school-based interventions have been designed and evaluated in Hong Kong to increase students’ physical activity levels during the school day, including physical education lessons [15]. However, few school-based interventions were not designed to enhance MVPA in the after school period or during weekends (e.g., [16]). Interventions involving parents may help to increase children’s physical activity during these time periods by expanding and extending opportunities to be active (e.g., [17, 18]).

### Children’s physical activity habits and parental influence

Children’s physical activity habits are shaped by their parents. This is particularly true for younger children who, compared with adolescents, require more care and spend more time with their parents. Parents typically act as the primary socialization agents in young children’s lives. Not surprisingly, researchers have found positive associations between parents’ and their children’s physical activity levels [13, 19, 20]. A meta-analysis conducted by Yao and Rhodes [21] found that support from parents and their modelling behaviors were related to children’s physical activity. Further, researchers have found that increases in joint parent-child physical activity may also be an effective method for increasing activity levels of both parents and children [22].

When compared to Australian parents, Hong Kong children report significantly lower levels of parental support for physical activity [13]. This may be due to Hong Kong parents’ lack of knowledge, or the diminished value they place on physical activity due to the relative importance of children’s academic achievement [23]. Contrary to these parents’ beliefs, researchers have shown that restricting children’s physical activity may be counterproductive to their academic performances [24]. For instance, researchers found that physical activity may improve children’s cognitive function [4, 5, 25] and academic performances [6]. Therefore, physical activity promotion programs in Hong Kong should involve parents, and educated them in or reinforce their understanding of the benefits of physical activity for their children’s cognitive and academic performance.

Parents and their children spend large portions of their time together in Hong Kong, especially during weekends. As such, there are ample opportunities for family-based physical activities. Given this situation, family-based physical activity programs are appropriate for the Hong Kong context. This is important because there is an association between the activity levels of parents and their children [19], as well as their attitudes towards physical activity [26]. Thus, increasing activity levels of both children and their parents may result in sustained changes in both generations, increasing the long-term effectiveness of the intervention. Researchers have shown that interventions that used a family-based approach can be effective [27]. Nonetheless, not all physical activity programs involving parents’ participation were effective [28]. New interventions must build on, and extend the extant literature to maximize intervention effectiveness. For instance, most family-based interventions focused on mothers only. Morgan and colleagues [17, 29, 30] found that father-focused interventions were also effective. The findings suggest that both mothers and fathers might be important social agents who affect intervention effectiveness. Nonetheless, to our knowledge, none of these programs was strongly theory-driven. Empirical evidence relating to the effectiveness of these programs is also unavailable.

### Physical activity and self-determination theory

Based on self-determination theory (SDT) [31], the manner in which support is provided, whether being need-supportive or controlling, could lead to different motivational or behavioral outcomes. For instance, a need supportive parent acknowledges children’s feelings and perspectives and provides meaningful choices; whereas a controlling one, uses pressure or rewards to initiate behavior [32]. According to the tenets of SDT, adopting a need supportive parenting style will promote higher levels of basic need satisfaction of competence, autonomy, and relatedness, and in turn positive behavioral change, and psychological well-being [31, 33]. By contrast, controlling behaviors may lead to the frustration or undermining of these basic needs, which may, in turn, diminish children’s physical and psychological well-being [34, 35]. Although empirical studies have been conducted to examine the latter relation in other domains such as physical education [36] and parenting [37], it has not been examined in parent-child dyads within the context of physical activity.

The “Active 1 + FUN” family-based physical activity intervention program for children and parents was designed to increase activity behaviors and fundamental movement skills of children, and also their fathers and mothers. We also aim to examine the relation between parents’ interpersonal styles and their children’s need satisfaction/frustration, and in turn the impact on physical activity behaviors and well-being. Through the proposed intervention, we also aim to help parents become more need supportive, and less controlling. The intervention will draw on previous evidence (e.g., [29, 30, 38]), but will also be tailored based on the sociocultural background of the Hong Kong context to enhance effectiveness. Specifically, children and parents who participate in the intervention will be invited to interactive workshops and activity sessions. Inexpensive and widely available sports equipment (e.g., skipping ropes, soft volleyballs, sponge flying discs) will be used during activity classes, and given to participants as gifts to encourage physical activity outside intervention classes. Interactive workshops will be provided to parents and children on topics such as healthy lifestyles, time management and parenting practices based on the tenets of SDT. One of the workshop goals is to provide a platform for parents to listen to children’s feelings about physical activity and provide suggestions on how parents can address children’s perspectives in a supportive way. Activity planning consultation, the parent-child physical activities homework and the available online materials will allow the participants to have a thorough understanding towards FMS and to set goals according to their abilities alongside to boost their physical activity level.

The effectiveness of the intervention will be evaluated using a randomized controlled trial. Eligible children and their parents will be randomly assigned to the experimental group or a wait-list control group. Quantitative measures will be taken at baseline, at the end of intervention period (accelerometer measure will be taken during a period that included the day of the tenth intervention session, secondary outcomes will be taken the week immediately before the session) and post-intervention follow-up (one year after baseline), for all children and their parents who took part in the intervention. The primary outcome of the trial will be time children’s spent in leisure-time MVPA, measured objectively using accelerometers.

### Objectives and hypotheses

The main objective of the current trial is to evaluate the effectiveness of a family-based physical activity intervention regarding changes in the objective measurement of the physical activity behaviors of parents and their children. We will also investigate the potential mediating effects of perceived parental autonomy support and controlling behaviors on children’s psychological need satisfaction or thwarting, and in turn their activity behaviors. Furthermore, we will examine how the intervention was perceived by the participants and whether it successfully initiated behavior changes through qualitative interviews. We hypothesize that relative to children and parents in the control group, participants in the experimental group will show significantly greater increases in MVPA from baseline to both post-intervention time points. We also hypothesize that children in the experimental group will report higher levels of perceived parental autonomy support, and lower parental controlling behaviors, when compared to their control group counterparts. Potential mediation effects of needs satisfaction and motivation on behavioral and health outcomes will be examined using structural equation modelling methods.

## Method

### Trial design

**Fig. 1:**
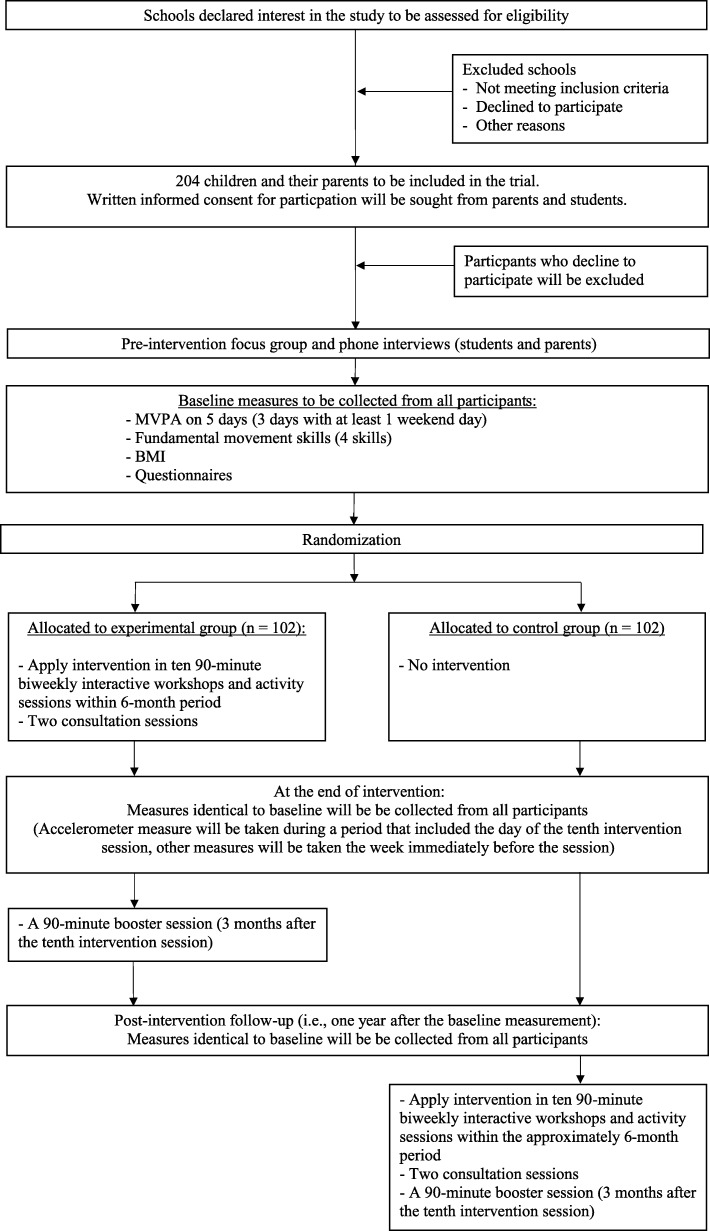
Flow diagram of the cluster randomized controlled trial

The “Active 1 + FUN” intervention will be evaluated using a randomized controlled trial. The trial was registered at ANZCTR (Id: ACTRN12618001524280) on 11 September 2018. The primary outcome will be objectively measured physical activity from children. A flow diagram of randomized controlled protocol is shown in Fig. 1.

### Sample size calculation

A power calculation was conducted to estimate the required sample size. Power calculation was based on the primary outcome of children’s MVPA, at the primary time point of 6-months. Calculations were conducted using G*Power 3.1.7. Based on the Healthy Dads, Healthy Kids study [29], intervention effect size was estimated at *d* = 0.5. With a Type I error probability of 0.05 and power of 0.8, the required sample size was calculated to be *N* = 128. To account for an estimated accelerometer non-compliance rate of 30% (i.e., cases with insufficient wear time) and a potential drop-out rate of 10% [14], a minimum of *N* = 204 children will be recruited.

### Participants

Students and their parents from Primary 3 to 5 (approximately 8 to 11 years old) will be recruited to take part in this study. A pool of gender-balanced participants will be recruited via eight local schools that meet our eligibility criteria. Specifically, schools will be eligible if they are (i) co-educational (i.e. mixed-sex); and (ii) either government primary schools or aided primary schools (i.e., schools that do not have an abundance of resources). We will aim to recruit at least one school from the three main regions in Hong Kong (i.e., Hong Kong Island, Kowloon or the New Territories) respectively. Invitation letters will be sent to randomly selected primary schools which meet the eligibility criteria. Before confirming their participation in the study, the Parent-Teacher Association (PTA) or school representatives will be invited to attend a briefing session to outline the background of the study. After confirmation of their participation, Primary 3 to 5 children and their parents will be recruited from schools. Approximately 25 to 30 families will be recruited from each school. Measurements of all outcomes will only be taken from participants who agree to participate in this study.

### Ethics, consent and permissions

Students and their parents will be required to provide written consent and complete the Physical Activity Readiness Questionnaire [39] to ensure they are physically healthy (i.e., no known episode of chest pain, dizziness, or joint problems after physical activity) to engage in MVPA before participating in this study. Parents will be required to sign informed consent forms if they agree to participate as a family unit. Ethical approval for the study was obtained from the Joint Chinese University of Hong Kong – New Territories East Cluster Clinical Research Ethics Committee (Ref: 2017.018).

### Procedures and randomization

Measures for primary and secondary outcomes will be taken at baseline, at the end of intervention period (accelerometer measure will be taken during a period that included the day of the tenth intervention session, secondary outcomes will be taken the week immediately before the session), and post-intervention follow-up (i.e., one year after the baseline measurement) to examine long-term intervention effects. After completing baseline measures, participants will draw a sealed envelope to determine whether they will be allocated to the experimental group or the wait-list control group. In each school, half of the recruited families will be allocated to the experimental group, and the other half will be allocated in the wait-list control group (i.e., 1:1 ratio). Participants allocated to the experimental group will receive ten bi-weekly intervention sessions over approximately 6 months immediately after baseline measures are taken. Participants allocated to the control group will be asked to attend measurement sessions, but they will only receive the intervention 12 months later (i.e., after the final follow-up time point). Each family will receive a HK$100 gift voucher for each completed assessment for the child and at least one parent. Intervention sessions will take place within school premises. All intervention sessions for a particular class will be led by the same activity leaders (two per session) throughout the intervention period. To encourage participation, each family will receive a transportation subsidy of HK$40 for the interactive workshops and activity sessions that they attend.

### Intervention

A relatively bottom-up approach will be adopted in designing and customizing the content of the activity sessions with reference to the fathers’, mothers’ and children’s perceived abilities (competence), the interests and the choices of physical activities (autonomy) respectively. Specifically, pre-intervention interviews will be conducted with potential participants of the study. Parent and children (five individuals per group, respectively) focus group interviews will be conducted in schools. Interviews will explore participants’ physical activity habits (e.g., “How much time did you spend on exercise or sport activities in the past 7 days?”), perceived competence (e.g., “Are you confident in doing the exercise[s]/sport[s] you have just mentioned?”, autonomy (e.g., “Can you please name 5 exercise[s]/sport[s] you like?”) and relatedness (e.g., “Can you share with me your experience of parent-child physical activities, exercise or sport activities? How do you feel? Why?”) regarding physical activity alongside the preferred types of parent-child physical activity (e.g., “If there are parent-child physical activity sessions provided, what kinds of physical activity do you want?”). Interviews with children and parents will be conducted in a quiet room within school premises. Phone interviews will be conducted with parents who are not able to attend the focus group interview during school-hours. The feedback received through these interviews will be used to fine-tune the final intervention components and contents.

The intervention also includes multiple components associated with specific behavior change strategies [40], and target specific theoretical constructs (see Table 1). The main intervention period will last for approximately 6 months, the length of intervention may vary among participating schools because of the differences of their academic calendars (exam periods, public or school holidays, and school activities would be avoided during the intervention to minimize disturbances to regular school activities). Participants will attend ten biweekly 90-min intervention sessions. Each session will consist of two parts, a 30-min interactive workshop followed by a 60-min activity session. An additional booster session will be provided approximately three months after the tenth intervention session. Both fathers and mothers will be invited to the intervention sessions, and at least one parent must be present during each intervention session.

**Table 1:** List of intervention components and associated dosage, behavior change strategies, and hypothesized mediators

Intervention components	Dose	Description	Behavior Change techniques using Michie et al.’s [40] taxonomy	Hypothesized mediators
1. Interactive workshop	Ten 30-min biweekly sessions	Storytelling will be the critical teaching strategy for the workshops. There will be different stories and themes for each of the workshop; for instance, the key concepts of SDT and the ‘SAAFE’ teaching principle, the importance and benefits of the routinizing physical activity. A strong focus will be on ways to increase parent’s need support and reduce controlling behaviors, in which the differences between supportive and controlling behaviors will be illustrated with real-life examples.	- Goals and planning - Feedback and monitoring - Social support - Shaping knowledge - Natural consequences - Repetition and substitution - Identity	- Need support/ controlling behaviors - Competence - Autonomy - Relatedness - Autonomous motivation
2. Activity session	Ten 60-min biweekly sessions	Parents and children will attend activity sessions together. Sessions include rough and tumble play, and FMS training.	- Feedback and monitoring - Social support - Shaping knowledge - Comparison of behavior - Repetition and substitution	- Need support/ controlling behaviors - Competence - Autonomy - Relatedness - Autonomous motivation
3. Parent-child physical activity homework	Weekly parent-child physical activity sessions outside the program (accumulate at least 30 min)	Parents and children are encouraged to plan, set goals regarding parent-child physical activities and spend at least 30 min (accumulative) per week to engage in any kind of physical activities together.	- Goals and planning - Feedback and monitoring - Social support - Repetition and substitution - Self-belief	- Competence - Autonomy
4. Activity planning consultation	Two 15-min session during intervention period	The MVPA and FMS measurement results and feedback will be sent to the participants via WhatsApp. A trained research assistant will help families identify gaps in time to fit in family activities, and overcome barriers they may have encountered.	- Goals and planning - Feedback and monitoring - Social support - Repetition and substitution - Self-belief	- Competence - Autonomy
5. Easy sports equipment	One set per family	A set of equipment including skipping rope, soft volleyball, sponge flying disc, etc.	- Antecedents - Self-belief	- Competence - Autonomy
6. Online materials	Participants can view the online resources with their gadgets any time anywhere they want.	Descriptions about the required FMS in various kinds of sports will be provided. Participants can easily make comparisons between different sports and reflect on their own strengths and weaknesses.		

Storytelling will be adopted as the fundamental teaching strategy in the interactive workshops. This strategy will help facilitate participants’ understanding of the following essential concepts: (i) tenets of SDT, (ii) benefits of routinizing physical activity, (iii) time management methods, and (iv) parenting practices. There will be different stories and themes for each of workshops, which will start with a short presentation to highlight the important message of the day. Then, each workshop will be followed by interactive activities and discussions between and within families. All video and written teaching materials for these sessions will be prepared by the research team. In line with SDT, one important goal of the workshops is to teach parents how to support their children’s basic psychological needs in various contexts and scenarios and avoid using external control. Role play activities will be embedded in workshops to enable learning and sharing in a more relaxed manner.

In the second part of the intervention sessions, parents and children will participate in physical activity together. These sessions will highlight the concepts of MVPA (i.e., huff and puff activity), FMS, motivation, and “co-physical activity”. There will be a gameplay period within the session, using innovative tools and methods, such as methods previously developed by Ha, Lonsdale, Lubans and Ng [41]. These will be followed by a skill development period using a set of easy sports equipment, including skipping rope, soft volleyball, sponge flying disc, which will be given to participants for free. The aim is to improve participants’ (children and parents) FMS proficiency. Researchers have found that these skills are important determinants of physical activity participation [11]. Hence improving such skills in children and parents may increase their future participation in physical activity beyond the intervention period. Both parents of children will be invited to take part in the intervention sessions. Specifically chosen sessions will be more tailored for mothers or fathers, respectively. The corresponding parent will be strongly encouraged to take part in these classes, even though they may be unable to attend other sessions.

Apart from the workshops and activity sessions, there will be weekly parent-child physical activity homework that will encourage participants to spend at least 30 min in total per week to engage in parent-child physical activity. Physical activity homework is an effective means to promote physical activity of students outside of school hours [42–44], as such parent-child physical activity homework will also promote the physical activity level of both parents and children. An activity logbook will be given to each family to encourage parent-child physical activity planning and to self-report and provide descriptions regarding the relevant parent-child physical activities they have done (e.g., date and time, with whom, the types of co-physical activity and duration). Also, each family will receive two 15-min consultation sessions during the intervention period. Essentially, each family will receive consultations on how to overcome barriers to engaging in physical activity as a family unit. They will receive scheduling help to identify time gaps for family activities. Families will be encouraged to begin a healthier lifestyle and will be prompted to provide reciprocal reinforcement (i.e., parents and children reinforcing each other simultaneously; [29]). Participants will be assisted to set short- and long-term goals for each family member during the consultations. A research assistant will be trained specifically to provide such consultations. Furthermore, online materials regarding the interactive workshops and FMS will be provided to all the participants. Descriptions about the required FMS in various kinds of sports will be provided. Participants can easily make comparisons between different sports and reflect on their own strengths and weaknesses. A booster session with fun physical activities will be provided to the participants approximately 3 months after the 6-month main intervention period.

### Activity leader training and fidelity of intervention

All intervention sessions will be facilitated by activity leaders, who are professionally trained activity instructors or physical education teachers. A train-the-trainer approach will be adopted; all activity leaders will receive two half-day training workshops led by the lead author and a co-author before the start of the intervention. All training materials, including presentation slides and videos of the workshop, will be uploaded to an online resource platform accessible to activity leaders. The unit plan of intervention sessions, and materials to be used in intervention sessions will be provided via the platform. The training workshops will be videotaped and uploaded to the online resources platform shared to the activity leaders. The activity leaders can revisit the scenarios and critical ideas anytime anywhere they want.

The tenets of SDT [31] and SAAFE teaching principles [45] will be introduced to activity leaders during training workshops. The activity leaders will be trained to apply the theory and teaching principles in all of the activity sessions. More specifically, the activity leaders will be trained to deliver sessions that are: (i) Supportive (i.e. the activity leaders should be supportive and exert their endeavors to promote positive interactions between parents and their children), (ii) Active (i.e. sessions should be highly active and with minimal transition time), (iii) Autonomous (i.e. sessions should involve opportunities for participants’ choice), (iv) Fair (i.e. all participants should be provided with opportunities to experience success), and (v) Enjoyable (i.e. sessions should be enjoyable with a variety of activities). As such, a practical session would be provided during the training. Ample time and opportunities will be given to the activity leaders to implement the aforementioned teaching techniques for different activities. There will be a follow-up small group discussion session immediately after the practical session, and the activity leaders can also ask questions and receive feedback from the lead author and a co-author. Further, activity leaders will be provided with a self-reflective questionnaire based on the SAAFE principles to complete after each intervention session. This process will help activity leaders to identify things they have done well and areas for improvement.

A half-day top-up training workshop will be provided to the activity leaders at the mid-point of the intervention to collectively reflect on their teaching experiences. This will provide them with an opportunity to share their barriers to implementation and potential solutions. To ensure the intervention delivered by each activity leader is comparable, all intervention sessions will be video-recorded and at least two sessions be reviewed and assessed with reference to a “SAAFE Principles Observation Checklist” (e.g., “Provide private rather than public feedback”, “involve participants in the creation and modification of activities and rules”). Activity leaders can also view the video-recorded sessions of their counterparts online, and they are encouraged to share their teaching experiences, discuss and comment on each other’s teaching to create a more mutually supportive environment among activity leaders.

### Primary and secondary outcomes of the trial

The primary outcome of the randomized controlled trial is children’s MVPA. Parents’ MVPA and the time parents and children spend doing activity together (co-physical activity) will be measured as secondary outcomes. Other secondary outcomes of the trial include (i) parents’ and children’s FMS competency, (ii) parents’ and children’s BMI, (iii) children’s perceived need support and control, (iv) parents’ provision of need support and psychological control, (v) children’s and parent’s basic psychological needs satisfaction and thwarting, (vi) parents’ motivation towards physical activity, (vii) children’s and parent’s well-being.

#### Moderate-to-vigorous physical activity – Children and parents

Children’s and parents’ time spent in daily MVPA over a five-day period will be measured using ActiGraph wGT3X-BT accelerometers worn at the waist. Using the criteria developed by Evenson, Catellier, Gill, Ondrak and McMurray [46] (for children) and Freedson, Melanson, and Sirard Freedson, Melanson, and Sirard [47] (for parents), the sum of daily minutes spent in moderate and vigorous activity will be used as a measure for MVPA. One-second epochs will be used for the calculation of MVPA [48]. Cases will be considered valid if participants wore the accelerometer for at least eight hours on a minimum of three days, with at least one weekend day. Using proximity sensors in the ActiGraph devices [49], the duration of co-physical activity periods per day will also be measured. Participants will be required to wear the devices throughout their waking hours within the five-day data collection period except during any water-based activities (e.g., water sports, bathing) or occasion where they are not allowed to wear the devices (e.g., a contact sports match).

#### Fundamental movement skills competency – Children and parents

Four FMS of children and parents will be assessed, namely, kick, catch, overhand throw, one-hand forehand strike. These skills will be assessed using the Test of Gross Motor Development-3 (TGMD-3) [50]. Using the protocol by Chan et al. [51], participants will first be shown a video demonstration of the skill to be assessed. Their performances with then be video-taped and assessed using the TGMD-3. The videos will be reviewed and rated by a trained research assistant with a degree in physical education.

#### Body mass index – Children and parents

Participants’ body height and weight will be measured to calculate their BMI. This outcome will be used as a pseudo-measure of their physical health.

#### Children’s perceived need support and control – Children only

The degree to which children perceive their parents to be need-supportive or controlling will be measured using an adapted version of Perceived Parental Autonomy Support Scale [52]. The adapted scale comprises of six items measuring need supportiveness (e.g., “Within certain limits, my parents allowed me the freedom to choose my own activities”) and seven items tapping psychological control (e.g., “When my parents wanted me to do something differently, they made me feel guilty”). Respondents will provide responses using a 7-point scale from 1 (“Do not agree at all”) to 7 (“Very strongly agree”).

#### Provision of need support and psychological control – Parents only

Parents will be asked to self-report the *frequency* in which they use need supportive or controlling strategies in relation to their children’s physical activity behaviors. Items will be adapted from the scale used to measure children’s perceived support. Example items include: “Within certain limits, I allowed my child the freedom to choose their own activities”, “When I asked my child to do something differently but he/she does not listen, I will make him/her feel guilty”. Responses will be made using a 5-point scale ranging from 1 (“Never”) to 5 (“Always”).

#### Basic psychological needs satisfaction and frustration – Children and parents

The satisfaction and frustration of three basic psychological needs of autonomy, competence, and relatedness have essential consequences for one’s physical and psychological health [33]. Children and parents’ perceived basic need satisfaction and frustration will be measured using a scale adapted from Ng, Ntoumanis, Thøgersen-Ntoumani, Stott, and Hindle [53]. The 18-item scale measures the facets of perceived basic need satisfaction or furstration towards physical activity participation; for instance, “People I know tell me I am good at sports” (competence satisfaction), “I feel free to choose what to do” (autonomy satisfaction), “people around me care for me” (relatedness satisfaction), “I encountered situations where I was made to be incompetent” (competence frustration), “I feel forced to follow decisions made for me” (autonomy frustration), and “I feel others dislike me” (relatedness frustration). Respondents will make responses using a 7-point scale from 1 (“Strongly disagree”) to 7 (“Strongly agree”).

#### Well-being – Children and parents

The Cantonese versions of the KIDSCREEN health-related quality of life questionnaire [54] will be used as a measure for children’s well-being. It is a ten-item questionnaire which assesses children’s physical well-being (e.g., “Have you felt fit and well?”), psychological well-being (e.g., “Have you felt sad?”), autonomy (e.g., “Have you been able to do the things that you want to do in your free time?”) and parent relation (e.g., “Have your parent[s] treated you fairly?”), peers and social support (e.g., “Have you had fun with your friends?”, and school environment (e.g., “Have you got on well at school?”). Respondents will make responses using a 5-point scale from 1 (“Never”) to 5 (“Always”). A Chinese version of the Flourishing Scale [55] will be used to measure parents’ psychological well-being. The scale consists of eight items which measure the facets of one’s psychological well-being; for instance, the positive relationships and having life meaning and purpose (e.g., “I am engaged and interested in my daily activities”). Respondents will respond using a 7-point scale from 1 (“Strongly disagree”) to 7 (“Strongly agree”).

### Blinding

Participants and activity leaders will not be blinded to group allocation, since only those allocated to the experimental group will attend the intervention sessions in the first year. Research assistants who are responsible for data collection will be blinded to group allocation at all time points.

### Statistical methods

Data analyses will be conducted using linear mixed models using an intention-to-treat approach. Group differences concerning all primary and secondary outcomes will be examined. Specifically, a two-level (i.e., level 1 = time, level 2 = individual) model will be examined for each outcome, using the equation: Outcome = B_0_ + B_1_*Time + B_2_*Group + B_3_*Time*Group. The B_1_ coefficient will be parameterized as a variable with random slope and random intercept at all levels. Additional analyses will be conducted by adding the potential covariates and mediators to the models. An intervention effect will be deemed to be present if the B_3_ coefficient was statistically significant from zero.

Structural equation modelling will also be used to test the structural model of children’s perceived need support/control predicting need satisfaction/thwarting, and in turn physical activity behaviors and well-being. In the same model, we will examine the relationship between parents’ self-reported need supportive/controlling behaviors and children’s perception of the same constructs. Based on the work of Radel, Sarrazin, Legrain, and Wild [56], we will also examine the link between parents’ need satisfaction and their parenting styles in the model. To account for the relatively small sample size, a Bayesian approach will be used for structural equation modelling analyses (e.g., [53]).

### Focus group interviews and data analyses

Using a mixed-method approach, semi-structured in-depth qualitative interviews in the format of focus group or individual interview will be conducted by a trained research assistant at different time points, namely, pre-intervention (information provided above), mid-intervention, post-intervention, and at follow-up (i.e., one year from start of intervention). While at least one participating school from the three main regions in Hong Kong (i.e., Hong Kong Island, Kowloon or the New Territories) respectively will be invited to participate in the interviews. Potential parent and student interviewees will be recruited with the assistance of the PTA and teacher representatives. Interview guides based on the SDT will be developed, for every interviewee’s answer, additional probes and follow-up questions will be posed. Field notes will be written during and immediately after the interviews.

At mid-intervention, parents and children will be interviewed respectively to understand their learning experiences, the performances of the activity leaders and the difficulties faced. The participants’ feedbacks will be used to improve the intervention in both the short-term and long-term. Specifically, selected feedback from these interviews will be presented to coaches during the top-up training workshop. Whereas the post-intervention and follow-up interviews with reference to the previous research done by Hassandra, Goudas and Chroni [57] will be conducted to generate more in-depth information (i.e., impacts of involving more physical activity alone and/or with family in future) regarding the experience of participating in the designed intervention. Interviewees will be asked questions about their perceptions of competence (e.g., the levels of FMS), autonomy (e.g., availability of choices), and relatedness (e.g., support from or experiences in playing with father and/or mother). Further, the interviewees will be asked to describe which types of supportive or controlling behaviors are most influential or effective. Interviews with children will be conducted within school premises immediately after the booster session while telephone interviews with parents will be completed within a similar period.

All interviews will be audio recorded and transcribed verbatim. After the interviews are transcribed, they will be sent to the participants to review, to check for accuracy (member checking) and establish credibility. Any necessary changes will be made. The transcribed raw contents will be set out in lists, and similar items will be grouped under representative themes. Initial codes will be created openly using NVivo version 10 (QSR International) to categorize transcripts into components that are of potential significance to the research objectives.

## Discussion

It is of paramount importance to help children to develop the knowledge, skills and motivation to lead an active lifestyle from a young age. Despite the fact that research has suggested that physical activity has numerous health benefits, inactivity in children is commonplace across the globe, and Hong Kong is not an exceptional case. As Hong Kong is a meritocratic city, parents often place a strong emphasis on their children’s academic performances and neglect the extensive benefits of physical activity [23]. Thus, it is not surprised that the school children in Hong Kong reported that they generally received limited parental support for physical activity participation [13].

To address the physical inactivity of children, we designed a scalable family-based intervention program to increase children’s and their parent’s MVPA during leisure time and promote an active lifestyle among families. As parents are one of the critical socialization and change agents, it is particularly vital to reinforce their understanding of the benefits of an active lifestyle and instill them into the optimal type of support for children’s physical activity; in order to promote both physical and psychological health within families. Our family-based intervention targets the behaviors of parents and children and parents’ parenting methods which may yield promising results regarding the family’s engagement in physical activity during leisure time.

Based on our knowledge, our study will be the first theory-driven and relatively bottom-up family-based intervention program specifically designed to tackle the inactivity of children in a local family context. The study is underpinned by facets of SDT, which aim to investigate the parent-child dyad interactions within a context of the physical activity that previous empirical studies have not examined. To empower participants more effectively, a relatively bottom-up approach will be adopted to customize the content of activity sessions concerning the participants’ perceived abilities (competence), interests and choices (autonomy), and experiences of parent-child physical activities (relatedness). Moreover, the mediating effects of perceived parental autonomy support and controlling behaviors on children’s psychological need satisfaction or frustration, and in turn their activity behaviors will be tested using structural equation modelling methods.

Through the proposed intervention, we also aim to help parents become more need supportive and less controlling. It is also critical to understand how the intervention was perceived by the participants and whether it successfully initiated behavior changes will be explored through qualitative interviews. We hypothesized that the intervention will increase parents and children’s leisure-time MVPA and the children will experience higher levels of perceived autonomy support and lower controlling behaviors. Important elements related to family-based activity programs will be identified. Activity leaders trained by the researchers will lead the intervention workshops and activity sessions. Therefore, if shown effective, these sessions could be organized and delivered by trained trainers in the future. Consequently, this intervention could be scaled to a larger number of Hong Kong schools in the future and could impact a broader population of schoolchildren.

## Abbreviations

BMI: Body mass index
FMS: Fundamental movement skills
MVPA: moderate-to-vigorous physical activity
SAAFE: Supportive, Active, Autonomous, Fair, and Enjoyable
